# Novel genetic linkage of rat *Sp6* mutation to Amelogenesis imperfecta

**DOI:** 10.1186/1750-1172-7-34

**Published:** 2012-06-07

**Authors:** Taro Muto, Keiko Miyoshi, Taigo Horiguchi, Hiroko Hagita, Takafumi Noma

**Affiliations:** 1 Department of Molecular Biology, Institute of Health Biosciences, The University of Tokushima Graduate School, 3-18-15, Kuramoto-cho, Tokushima, 770-8504, Japan; 2 PRESTO, Japan Science and Technology Agency (JST), Saitama, Japan; 3 Laboratory for Stem Cell Biology, RIKEN Center for Developmental Biology, 2-2-3, Minatojima-minamimachi, Chuo-ku, Kobe, 650-0047, Japan

**Keywords:** Amelogenesis imperfecta, AI, AMI, Animal disease model, Autosomal recessive, *Sp6*

## Abstract

****Background**:**

Amelogenesis imperfecta (AI) is an inherited disorder characterized by abnormal formation of tooth enamel. Although several genes responsible for AI have been reported, not all causative genes for human AI have been identified to date. AMI rat has been reported as an autosomal recessive mutant with hypoplastic AI isolated from a colony of stroke-prone spontaneously hypertensive rat strain, but the causative gene has not yet been clarified. Through a genetic screen, we identified the causative gene of autosomal recessive AI in AMI and analyzed its role in amelogenesis.

****Methods**:**

cDNA sequencing of possible AI-candidate genes so far identified using total RNA of day 6 AMI rat molars identified a novel responsible mutation in *specificity protein 6* (*Sp6*). Genetic linkage analysis was performed between *Sp6* and AI phenotype in AMI. To understand a role of SP6 in AI, we generated the transgenic rats harboring *Sp6* transgene in AMI (*Ami*/*Ami* + *Tg*). Histological analyses were performed using the thin sections of control rats, AMI, and *Ami*/*Ami* + *Tg* incisors in maxillae, respectively.

****Results**:**

We found the novel genetic linkage between a 2-bp insertional mutation of *Sp6* gene and the AI phenotype in AMI rats. The position of mutation was located in the coding region of *Sp6*, which caused frameshift mutation and disruption of the third zinc finger domain of SP6 with 11 cryptic amino acid residues and a stop codon. Transfection studies showed that the mutant protein can be translated and localized in the nucleus in the same manner as the wild-type SP6 protein. When we introduced the CMV promoter-driven wild-type *Sp6* transgene into AMI rats, the SP6 protein was ectopically expressed in the maturation stage of ameloblasts associated with the extended maturation stage and the shortened reduced stage without any other phenotypical changes.

****Conclusion**:**

We propose the addition of *Sp6* mutation as a new molecular diagnostic criterion for the autosomal recessive AI patients. Our findings expand the spectrum of genetic causes of autosomal recessive AI and sheds light on the molecular diagnosis for the classification of AI. Furthermore, tight regulation of the temporospatial expression of SP6 may have critical roles in completing amelogenesis.

## **Background**

Amelogenesis imperfecta (AI) is a genetic disorder characterized by morphological and functional defects of tooth enamel formation. AI disease entity has been classified into four types: hypoplastic, hypocalcified, hypomaturation, and hypomaturation-hypoplastic with taurodontism based on the clinical symptoms and hereditary modes [1,2]. Several genes responsible for AI such as *AMELXAMBNENAMMMP20KLK4DLX3WDR72FAM83H*, and *FAM20A* have been so far reported [3-6]. Mutations of *AMELX* cause X chromosome-linked AI. Mutations of *AMBNENAMDLX3*, and *FAM83H* cause autosomal dominant AI. For autosomal recessive AI, mutations of *ENAMMMP20KLK4*, WDR72, and *FAM20A* have been demonstrated as shown in Table 1. However, not all causative genes for AI-related disease have been identified to date [5-8].

**Table 1:** Causative genes of amelogenesis imperfecta

**Inheritance pattern**	***Gene***	**Human chromosome**	**Rat chromosome**	**Mutation in AMI**	**Reference in human**
X-linked	*Amelx*	X	X	*n.d.*^*a*^	[9-21]
Autosomal dominant	*Ambn*	4	14	*n.d.*	*n.d.*
	*Enam*	4	14	no	[14,22-27]
	*Dlx3*	17	10	no	[28]
	*Fam83h*	8	7	*n.d.*	[29-34]
Autosomal recessive	*Enam*	4	14	no	[35,36]
	*Mmp20*	11	8	no	[26,37-39]
	*Klk4*	19	1	no	[40]
	*Wdr72*	15	8	no	[41]
	*Fam20a*	17	10	no	[3,4]

^*a*^*n*.*d.*: not determined.

**Figure 1:**
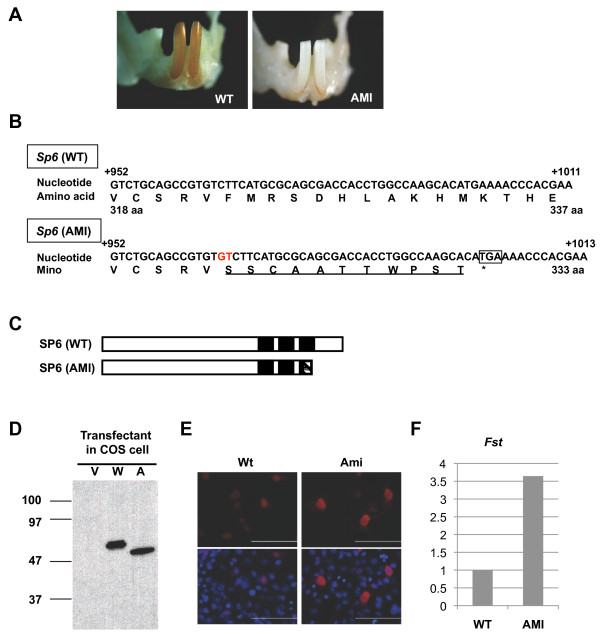
**Identification of a 2-bp insertion in*Sp6* in AMI rats. A.** Representative phenotype of WT and AMI rats. Photographs show mandibular incisors from each of the rats. **B.** The nucleotide sequence of *Sp6* in wild-type (WT) and AMI rats. The 2-bp insertion is highlighted (red). The square represents the premature stop codon. The amino acid sequence deduced from the codons is shown below the nucleotide sequence. The altered amino acid sequence in Ami-SP6 is underlined. **C.** Schematic representation of SP6. The N-terminal region is located to the left. Black boxes represent the position of the zinc finger motifs. The box with diagonal lines shows the altered amino acid sequence in Ami-SP6. **D.** Western blot analyses. COS7 cells were transfected with an expression vector only (V) or with FLAG-tagged *Wt*- (W) or *Ami*- (A) cDNA. Samples were blotted with FLAG antibodies. **E.** Subcellular localization of FLAG-tagged SP6 by immunocytochemical analysis. Red, FLAG-tagged protein; Blue, Nuclei. **F.** Relative expression levels of *Fst* in WT and AMI molars.

Forward genetics using animal disease models is a powerful approach to identify novel pathogenic genes responsible for the disease, and to elucidate the molecular basis of gene functions. In this study, we focused on AI using a disease model, AMI, which has been reported as a spontaneous AI mutant rat with chalky white teeth isolated from a colony of stroke-prone spontaneously hypertensive rat (SHR-SP) strain [42] (Figure 1A). In general, the surface of incisor enamel (solely on the labial side) in rodent shows yellowish-brown color. The molecular mechanisms of pigmentation are not yet clear, however, it is observed to store the irons with ferritin in the ameloblasts at pigmentation stage [43]. The iron-ferritin complex is transferred to lysosomes for degradation. Then, metabolically processed iron is released from ameloblasts and deposited on the surface of enamel matrix as a yellowish-brown layer. The whitish appearance of incisors indicates these processes are not complete, in terms of the defects of ameloblast differentiation, and resulting the enamel defect. The original SHR-SP strain, hereafter referred to as WT strain, does not have any AI phenotype. The AI phenotype in AMI is hypoplastic, and inherited as an autosomal single recessive trait; however, the causative gene has not been determined yet [42]. In this study, we clarified the genetic causes of autosomal recessive AI in AMI, and dissected the possible function of SP6 in amelogenesis.

## **Methods**

### **Animals**

Animal experiments were approved by the Ethics Committee for Animal Experiments of the University of Tokushima (No.06105). Both SHR-SP and AMI rats were supplied from Daiichi Seiyaku, Co., Ltd. and maintained at the animal facility in the University of Tokushima. To investigate whether the *Sp6* transgene can rescue the AI phenotype in AMI rats, we crossed *Sp6* transgenic (Tg) rats, which we generated previously [43], with AMI rats to generate a *Sp6* transgene homozygous mutant (*Ami*/*Ami* + *Tg*), and used for further analyses.

### **cDNA sequencing of AI candidate genes and*Sp6***

The coding regions for *Sp6* and other candidate genes were amplified from molar cDNA samples from WT and AMI rats by PCR using the primers shown in Table 2 with KOD plus DNA polymerase (TOYOBO, Osaka, Japan) or PrimeSTAR HS DNA polymerase (Takara, Shiga, Japan). PCR products were cloned into the pGEM-T easy vector (Promega, Madison, WI). The sequences of the products were analyzed with an ABI PRISM 3100-Avant Genetic Analyzer (Applied Biosystems, Foster City, CA).

**Table 2:** Gene-specific primers for coding regions

***Gene***	**Primer sequence**
*Dlx3*	5′-CCAGCATGAGCGGCTCCTTCGATCGCAAGC-3′
	5′-GGTACTCAGTACACAGCCCCAGGGTTAGGC-3′
*Dlx4*	5′-CCGCAATGACCTCTTTACCCTGTCCCCTTCC-3′
	5′-CAGACTCACATCCTCTGAGGCAGCGCCAGC-3′
*Enam*	5′-AATAAATGTGTCTTGCTCCTTGGCTCTCTG-3′
	5′- AATACCTAAGCCTGAAGCAGTAAACAGCCG-3′
*Fam20a*	5′-GGGCCATGCCCGGACTGCGCAGGGACCGCC-3′
	5′-TTGCTGCCGTTAGCTTGTCAGATTAGCCTG-3′
*Klk4*	5′-CCAATATGATGGTCACTGCACGAACCCCC-3′
	5′-CAGAGCTATCTTGTCTGAATGGTGGTCCAG-3′
*Mmp20*	5′-AGGAGATGAAGGTGCTACCTGCCTCTGGCC-3′
	5′-ACGATTCAGCAACCAACCCAGGAGCTGG-3′
*Ngfr*	5′-GTGCAATGAGGAGGGCAGGTGCTGCCTGCAG-3′
	5′-TGAGTTCACACTGGGGATGTGGCAGTGGAC-3′
*Sp2*	5′-GCAACATGACGCTCACCCTGCCACTCAACAACC-3′
	5′-TGGCCTTACAAGCCCTTCGTGCCTAGGTGGGTC-3′
*Sp6*	5′-CCGGCAATGCTAACCGCTGTCTGTG-3′
	5′-GGCTCAGTTGGAGGACGCCGAGCTG-3′
^*a*^*Wdr72(a)*	5′-CTAACATGAGGAGTGCTCTGCAGGCTGTGG-3′
	5′-TGCGCATCATCGCCGCAGTT-3′
^*a*^*Wdr72(b)*	5′-GCGTGCCCGGAAGCACCTTT-3′
	5′-TCGGGACTCCATCCATCTAGCAC-3′

^*a*^*Wdr72*: We divided the coding region into two fragments (a and b) for cloning.

### **Genotyping**

Genomic DNA was isolated from the tail tips of the rats and subjected to PCR analysis using the following four primers in a single tube. For WT *Sp6* amplification, WT-Sp6.F-CTT (5′-GTC TGC AGC CGT GTC TT-3′), rSp6.genomeR (5′-CTG GCA GCC TAA ATA ATA TTC AAG CAG-3′), GAPDH-S (5′-CAT TGA CCT CAA CTA CAT GG-3′), and GAPDH-AS (5′-CTC AGT GTA GCC CAG GAT GC-3′) were used, and for AMI *Sp6*, AMI-Sp6.F (5′-GTC TGC AGC CGT GTG TC-3′) was used instead of WT-Sp6.F-CTT. Primer concentrations were as follows: *Sp6*, 0.75 mM; *Gapdh*, 0.25 mM. *Sp6* (344 and 346 bp for WT and AMI, respectively) and *Gapdh* (722 bp) were amplified using a thermal cycler (94 °C for 4 min, 33 cycles of 94 °C for 30 s, 63 °C for 30 s, 72 °C for 30 s, and an extension step at 72 °C for 7 min) using Taq DNA polymerase (Promega).

### **Western blot analysis and immunocytochemistry**

COS7 cells were transfected with a pCI-neo mammalian expression vector (Promega) carrying FLAG-tagged rat *Sp6* (WT or AMI) or a mock vector control. Immunodetection and immunocytochemical analysis were performed as described previously [44].

### **Immunohistochemistry**

Immunohistochemical analysis of postnatal day 1 incisors was performed as described previously [44]. For longitudinal sections of adult incisors, craniofacial regions were isolated from adult rats after intracardiac perfusion with phosphate-buffered saline containing 5 U/ml heparin followed by fixation with 0.1 M phosphate buffer (pH 7.4) containing 4 % paraformaldehyde. Postfixation was performed by immersion of the dissected specimen into the same fixative for 2 h. Postfixed samples were decalcified in 10 % formic acid–4 % citric acid decalcification solution for 2 weeks or more at 4 °C. Decalcified maxillae were trimmed and divided into three pieces vertically to the longitudinal axis of the incisors, as described previously [43,45]. Each incisor segment was embedded in paraffin, and a series of longitudinal sections (6-μm thick) were prepared. The sections were treated with 3 % hydrogen peroxide in methanol for 20 min and boiled in antigen-unmasking solution (Vector, Burlingame, CA, USA) using a microwave. For immunostaining, samples were blocked with 3 % horse serum in phosphate-buffered saline (−), incubated with rabbit anti-rat SP6 antiserum [44] or normal rabbit serum as primary antibodies (1:400), and incubated with Histofine Simple Stain Rat MAX-PO (R) (Nichirei, Tokyo, Japan). DAB-buffer tablets (Merck, Darmstadt, Germany) were used to visualize signals. Sections were slightly cross-stained with hematoxylin and mounted in Entellan New (Merck).

### **Histological analysis**

Deparaffinized longitudinal sections of the maxillary incisors (12 μm) were stained with hematoxylin for histological analysis. The ameloblast differentiation stages were determined from the apical to the incisal end of the incisors using a modification of Warshawsky and Smith’s classification [46]. Presecretory ameloblasts face pulp or dentin, and the secretory stage starts from the position where the enamel matrix appears. The maturation stage starts from the point where the ameloblasts rapidly begin to decrease in size. In the reduced stage, ameloblasts become cuboidal in shape without a cytoplasmic region above the nucleus. Images of the longitudinal sections of hematoxylin-stained incisors were captured under a light microscope connected to a CCD camera. The length of the ameloblast layer at each stage was measured using the ImageJ software (National Institutes of Health, Bethesda, MD, USA).

### **Macroscopic observation**

Incisors (7- to 9-week-old), digits, and whiskers (5- to 8-month-old) were recorded with a digital camera. The whiskers of newborn pups were observed under a MZ16 stereomicroscope (Leica Microsystems, Wetzlar, Germany).

### **Linkage analysis**

Sixty-two heterozygous (*Wt*/*Ami*) and 56 homozygous (*Ami*/*Ami*) mutant rats were examined to determine the correlation between AI and *Ami*. The JoinMap 4 software (Kyazma, Wageningen, Netherlands) was used for genetic linkage analysis.

### **X-ray analysis**

Radiographs of rat teeth were obtained using MCT-CB100MF (Hitachi Medico, Tokyo, Japan). The X-ray analysis system was operated at a 50 kV accelerating voltage with a 100 mA probe current.

## **Results and discussion**

To explore the causative gene of AMI, we first examined the sequences of the coding region of all reported causative genes for autosomal recessive AI, such as *Enam*, *Mmp20*, *Klk4*, *Wdr72*, and *Fam20a* using total RNA of day 6 AMI rat molars and compared these with those of WT rats. We did not find any mutations among them (summarized in Table 1), suggesting that a novel gene may cause AI.

We next examined the *Sp6* gene, which has been identified as a key player in tooth development [47,48]. SP6 is a member of the SP/KLF family of transcription factors, which have three tandem zinc finger domains of the classical C2H2 type at the C-terminal region [48,49]. Loss-of-function analyses demonstrated that deficiency of *Sp6* in mice leads to malformation of teeth, hair, digits, and lungs [50,51]. In particular, tooth anomalies are observed including supernumerary teeth (hyperdontia), abnormal dentin crystal structure, and enamel agenesis [50,51]. In addition, our *in vitro* gain-of-function studies of SP6 identified several downstream target genes in the dental epithelial cell line [44,52]. These findings indicate that SP6 may play important roles in ameloblast differentiation. When we performed the sequence analysis of *Sp6*, we found a 2-bp insertion between the nucleotide positions 965 and 966 in the coding region of *Sp6* in AMI rats (Figure 1B). This insertion caused a frameshift mutation with the addition of 11 cryptic amino acid residues from amino acid position 323 of SP6, disrupting the third zinc finger domain of SP6 (Figure 1C). To confirm whether the inserted *Sp6* (*Ami*) could be translated into a protein in cells, we transduced a CMV promoter-driven FLAG-tagged *Wt* (wild type-Sp6) or FLAG-tagged *Ami* expression vector into COS7 cells. The anti-FLAG antibody could detect both the Wt and Ami-type SP6 protein on western blot analysis (Figure 1D). Furthermore, subcellular localization of Ami-SP6 was detected mainly in the nuclei and, to some extent, in the cytosol, similar to Wt-SP6 (Figure 1E). Furthermore, we examined the expression level of *follistatin* (*Fst*) mRNA in postnatal day 6 (d6) molars from WT and AMI rats, because we previously demonstrated that *Fst* is one of the SP6 downstream target genes [44]. *Fst* expression was higher in AMI molars than in WT molars (Figure 1F). These results demonstrated that Ami-SP6 can be translated and located in the same manner as Wt-SP6, but it has different effects on downstream target gene expression.

**Figure 2:**
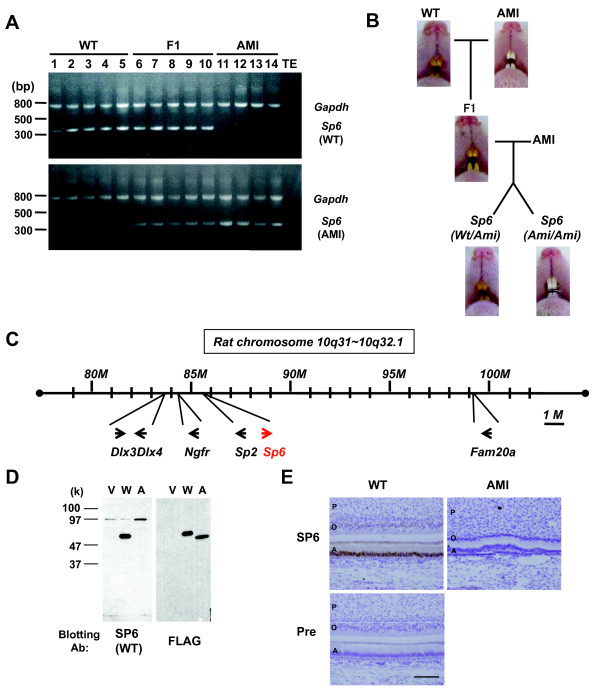
**Linkage analysis between*Sp6* and AI in AMI rats. A**. Genomic PCR products amplified by WT- (upper panel) or AMI- (lower panel) *Sp6*-specific primers. Sample numbers and the characteristics of the animals are denoted above the gel image. **B.** The color of the incisors was examined. Pups derived from the cross between F1 and AMI rats were sorted by the Sp6 genotype to determine the correlation between *Sp6* and AI in AMI rats (*Wt*/*Ami*, n = 62; *Ami*/*Ami*, n = 56). **C.** Schematic diagram of focal gene localization in rat chromosome 10q31–10q32.1. **D.** Western blot analyses. COS7 cells were transfected with expression vector only (V) or with FLAG-tagged *Wt*- (*W*) or *Ami*- (A) cDNA. Samples were blotted with the indicated antibodies. **E.** Immunohistochemical analysis of incisors from newborn pups. Sections from secretory stage incisors of WT rats and those of the corresponding region from AMI rats were stained with the anti-Wt-SP6 antiserum (SP6) or preimmune rabbit serum (Pre). Signals were obtained from DAB (brown). Sections were counterstained with Hematoxylin. Scale bar: 100 mm. A, ameloblasts; O, odontoblasts; P, pulp.

To determine whether the 2-bp insertion in *Sp6* correlates with the AI phenotype in AMI rats, we performed a linkage analysis. F1 rats were backcrossed with AMI to obtain descendants carrying *Ami* either heterozygously or homozygously. Although the color of the incisors in the first backcross generation was yellowish brown when the rats were heterozygotes with both *Wt* and *Ami*, *Ami* homozygote rats had chalky white incisors (Figure 2B). The *Sp6* genotypes in WT, AMI, and their F1 hybrids rats were analyzed by genomic PCR (Figure 2A), and we confirmed that all AMI rats were homozygous for *Ami*. Linkage analysis with 62 heterozygous (*Wt*/*Ami*) and 56 homozygous (*Ami*/*Ami*) mutant rats revealed that the AI phenotype and *Ami* homozygosity were strongly linked with a LOD score of 35.46.

*Sp6* is located in rat chromosome 10q31, and this locus is well conserved in human chromosome 17q21. There are several tooth-related genes and family member genes within 2 Mb of *Sp6* (Figure 2C). *Sp2* is a member of the same family as *Sp6*[49]. *Dlx3* has been reported as a causative gene for autosomal dominant AI [6], and *Dlx4* is located next to *Dlx3*[53]. *Ngfr*, also known as *p75NTR*, has been reported to be important for early ameloblast differentiation [54]. Since we found a strong link between this locus and the AI phenotype associated with Ami, we further analyzed these genes by cDNA cloning and sequencing in order to eliminate candidate AI causative genes. We could not find any mutations in *Sp2Dlx3Dlx4*, or *Ngfr* genes using d6 AMI molar RNA as a result (data not shown).

In order to distinguish Wt-SP6 from Ami-SP6, we prepared rabbit polyclonal antibodies against specific peptides for Wt-SP6 (amino acid residues 362–376) [44]. The specificity of the antibodies was confirmed using cell lysates of COS7 cells transfected with FLAG-tagged *Wt* or FLAG-tagged *Ami* by western blot analysis (Figure 2D). Next, we examined endogenous SP6 localization in the mandibular incisors of WT and AMI newborn pups by immunohistochemical analysis. In WT incisors, SP6 was detected in the nuclei of early secretory ameloblasts and contralateral odontoblasts, whereas no signal was observed in AMI incisors (Figure 2E).

**Figure 3:**
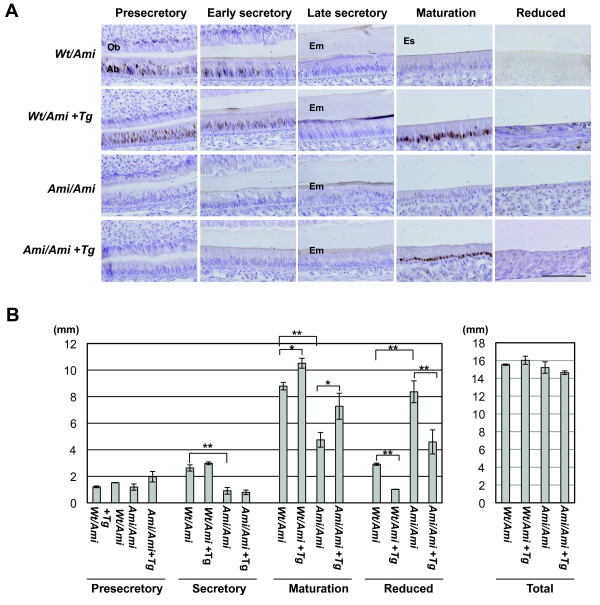
**Analyses of longitudinal sections of the maxillary incisors of 6-week-old rats. A**. Immunohistochemical analysis. Incisor sections were immunostained with antiserum against rat *Wt*-SP6. The sections were prepared from rats heterozygous (*Wt*/*Ami*) or homozygous (*Ami*/*Ami*) for mutant *Sp6*. Animals were sorted based on the transgenic *Sp6* (Tg) genotype. Scale bar, 100 mm. Ab, ameloblasts; Em, enamel matrix; Es, enamel space; Ob, odontoblasts. **B**. The length of the ameloblast layer for the indicated differentiation stages. Columns represent the average of two to three independent samples; bars indicate standard deviation. Statistical significance was evaluated by unpaired *t* tests for an indicated set of data. *p < 0.05, **p < 0.01.

Ameloblast differentiation is morphologically classified into presecretory, secretory, and maturation stages [46,55]. In this classification, the reduced stage is treated as a substage of the maturation stage. In order to verify the functional relationship between AI and SP6 protein in detail, we divided ameloblast differentiation into five stages: presecretory; early secretory; late secretory; maturation; and reduced. We then analyzed the localization of the SP6 protein in the ameloblasts of maxillary incisors from 6-week-old rats (Figure 3A). In *Wt*/*Ami* rats, SP6 signals were strong in the presecretory and early secretory ameloblasts and weak in the contralateral odontoblasts. SP6 signals were not detected in late secretory, maturation, and reduced stage ameloblasts (Figure 3A, top panels). On the other hand, homozygotes with the mutant *Sp6* (*Ami*/*Ami*) did not express the WT-SP6 protein at all, and defective ameloblast morphology was observed, such as shortened cells with immature polarization compared to the corresponding regions in *Wt*/*Ami* rats (Figure 3A, third panel).

To investigate whether the *Sp6* transgene can rescue the AI phenotype in AMI rats, we then crossed *Sp6* transgenic (Tg) rats with AMI rats to generate a *Sp6* transgene homozygous mutant (*Ami*/*Ami* + *Tg*). The *Sp6* Tg rat model was recently established by the introduction of a human CMV immediate early enhancer/promoter-driven *Sp6* coding sequence into Sprague–Dawley rats [43] Since tooth development is also driven by epithelial-mesenchymal interaction, we have used CMV enhancer/promoter to expect SP6 expression in both ameloblasts and odontoblasts. In *Ami*/*Ami* + *Tg* rats, the morphology of ameloblasts was similar to that observed in *Wt*/*Ami* rats from secretory to maturation stages (Figure 3A, fourth panel). The ameloblasts in *Ami*/*Ami* + *Tg* rats were aligned, well polarized, and their cellular height was restored to the levels similar to those in *Wt*/*Ami* rats (Figure 3A, top panels). However, tooth color, enamel calcification, and enamel matrix expression could not be rescued (data not shown). Subsequently, we examined SP6 expression in *Ami*/*Ami* + *Tg* rats. We could not detect any Wt-SP6 protein in the regions corresponding to the presecretory, secretory, and reduced stages in *Ami*/*Ami* + *Tg* rats, except for exclusive expression in maturation stage ameloblasts (Figure 3A, fourth panel). Consistent with these results, Wt-SP6 signals were detected in maturation stage ameloblasts, in addition to both presecretory and secretory ameloblasts, in the *Sp6* transgene heterozygous mutants (*Wt*/*Ami* + *Tg*) (Figure 3A, second panel). One of the possible reasons for the failure to rescue the AI phenotype is the silencing of *Sp6* transgene expression at the correct time and region because we recently found that SP6 is a short-lived protein (*t*_*1*/*2*_ ; 40 min) under the control of a proteasome pathway [52] and epigenetic mechanisms [56].

Next, we measured the length of the ameloblast layer at each differentiation stage using longitudinal sections of the maxillary incisors to determine whether there was a correlation between ameloblast differentiation and SP6 function (Figure 3B). In *Ami*/*Ami* incisors, the length of the ameloblast layer at the secretory and maturation stages was shorter, and the reduced stage was elongated compared with the *Wt*/*Ami* incisors (Figure 3B). In *Wt*/*Ami* + *Tg* and *Ami*/*Ami* + *Tg* rat incisors, we found that the length of the maturation stage ameloblast layer was longer than that of the non-Tg controls *Wt*/*Ami* and *Ami*/*Ami*, but the length of their reduced stage was much shorter compared with that of the same non-Tg controls. These results suggested that SP6 expression regulates cell morphology by an unknown mechanism. However, further analyses are necessary to confirm the molecular mechanism.

**Figure 4:**
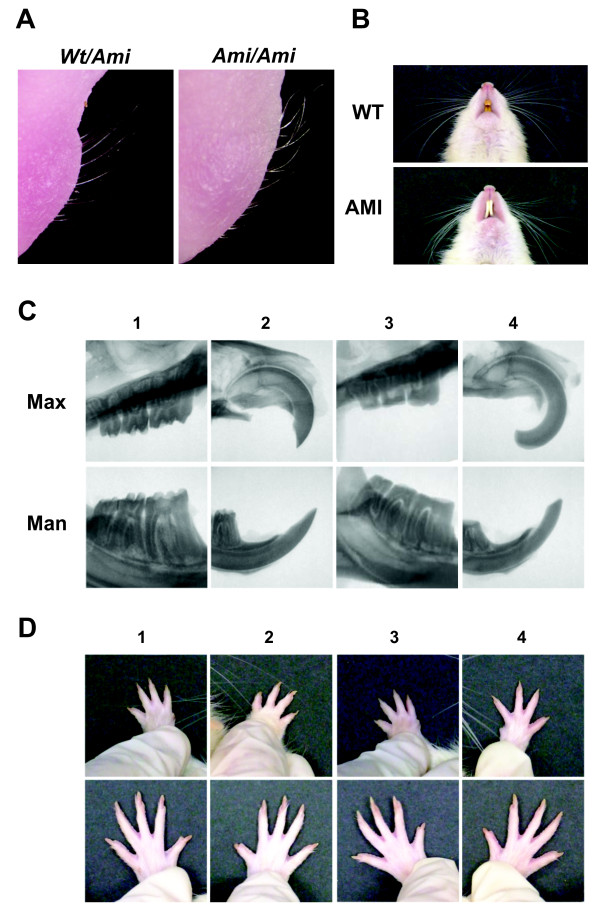
**Organogenesis requiring*Sp6* activity. A**. Whiskers of rats at postnatal day 1. Each *Sp6* genotype in the 1st backcrossed generation was examined. (*Wt*/*Ami*, n = 13; Ami/Ami, n = 21) **B**. Whiskers of adult rats. WT and AMI rats were examined when they were 6–8 months old (WT, n = 3; AMI, n = 14) **C**. X-ray analysis of the maxillae (Max) and mandibles (Man) of WT (1, 2) and AMI (3, 4) rats. Molars (1, 3) and incisors (2, 4) are highlighted (WT, n = 2; AMI, n = 2) **D**. Normal number of digits in AMI rats. Digits of the left (1, 3) and right (2, 4) limbs of WT (1, 2) and AMI (3, 4) rats are shown. Upper panels, forelimb; lower panels, hindlimb (WT, n = 3; AMI, n = 14).

*Sp6*-deficient mice have several developmental abnormalities such as hyperdontia (supernumerary), enamel agenesis, oligodactyly, syndactyly, and hairless phenotypes [50,51,57]. Therefore, we carefully examined other organogenesis processes in AMI rats that required SP6. The whiskers were present, but curly, in newborn AMI rats, whereas they were straight in WT rats (Figure 4A). In adulthood, the whiskers were feeble in AMI rats, but not in WT rats (Figure 4B). The number of teeth and their structure in both WT and AMI rats were normal as judged by X-ray analyses, except for enamel formation in AMI rats (Figure 4C). Because of this enamel defect, the incisors of AMI showed less-sharp ends of incisor tips and elongation of incisor length due to impossible grinding and continuous growing.

Furthermore, we observed normal numbers and morphology of the digits of both the forelimbs and hindlimbs of AMI and WT rats (Figure 4D). Basically, the AMI model shows only the AI phenotype. We summarized the phenotypic comparison between AMI rats and *Sp6*-deficient mice in Table 3.

**Table 3:** **Abnormalities in AMI rats and*Sp6*-deficient mice**

**Organ**	**Category**	**AMI rats** this study, [42,58]	***Sp6*-deficient mice** [50,51,57]
Teeth	Number	Normal	Excess (hyperdontia)
	Enamel	Thin, calcified	Absence or thin
	Ameloblasts		
	- Nuclear	Polarized	Not well-polarized
	-Tome’s process	No	Atypical
	-Morphological differentiation	Early regression	Defect
Hair		Normal	Short, alopecia, no fur
Whisker	Pups (at birth)	Curly	Rare, short, curly, alopecia
	Adult	Feeble	Delayed appearance
Digits		Normal	Syndactyly, oligodactyly

Recently, there was a notable report of a frameshift mutation of *Sp7*/*Osterix* in a patient that caused a similar disruption of the third zinc finger domain with additional amino acids and resulted in autosomal recessive osteogenesis imperfecta (OI) [59]. The known OI causative genes are restricted to type I collagen metabolic pathway, while *Sp7*/*Osterix* is a member of the SP/KLF family, which plays an essential role in osteoblast differentiation as a downstream target of RUNX2 [60]; therefore, it was an unexpected finding that a *Sp7*/*Osterix* mutation could cause OI. Whereas *Sp7*/*Osterix*-deficient mice could not survive [60], the OI phenotype of the frameshift mutant is far milder. Our study revealed a similar relationship between the *Sp6* frameshift mutation and AI as that between the *Sp7* frameshift mutation and OI. So far, there have been no other reports of the association between an *Sp6* mutation and AI patients, but our findings could encourage additional surveys of patients with AI of an unknown cause, particularly an autosomal recessive type of AI.

SP6 has three tandem zinc finger domains of the classical C2H2 type at the C-terminal region, which is common among SP/KLF family members [48,49]. Each finger recognizes the specific nucleotide sequences and contributes to the binding strength to DNA. In addition, the finger plays a role of the interface with the regulatory protein(s) [61]. SP1 is a well-characterized member of the SP/KLF family, and all three zinc fingers are essential for binding to importin alpha and contribute to nuclear localization [62]. Here, we identified a novel frameshift mutation caused by a 2-bp insertion in the *Sp6* coding sequence, resulting in disruption of the third zinc finger domain with an addition of an unrelated 11 amino acids (Figure 1B,C). This SP6 mutant form could enter the nuclei (Figure 1E). Our results indicate that the SP6 mutant form may have the following effects: 1) decrease in the affinity of DNA binding, and either loss or reduce of SP6 function as a transcription factor (Figure 1F); 2) decrease in the recruitment of interacting molecule(s), and a change in the gene expression pattern; 3) a novel recruitment or disappearance of protein–protein interactions through the cryptically added 11 amino acids; 4) a dominant-negative effect by chelating the regulatory domain interacting protein(s) at the N-terminal region of SP6; and 5) differential effects induced by the *Sp6* antisense transcript. Further investigations are necessary to confirm the possible effects induced by the SP6 mutant and understand the molecular basis for amelogenesis.

Amelogenesis imperfecta is an inherited disorder of enamel formation with both clinical and genetic heterogeneity [1,2]. In many cases, the nomenclature of the inherited diseases has been developed based on the phenotypes and inheritance patterns. Therefore, it is difficult to distinguish the highly variable clinical phenotypes. Recently, molecular basis of these inherited diseases are reorganized based on the phenotype-genotype relationship. Online Mendelian Inheritance in Man (http://www.ncbi.nlm.nih.gov/omim) is a well-organized and updated genetic disease database. However, there is very limited description about the autosomal recessive form of hypoplastic AI in the section of amelogenesis imperfect. Here, we demonstrated the novel genetic linkage between SP6 frameshift mutation and autosomal recessive type of AI using a spontaneous AI animal model. We presents the evidence for a novel genotype–phenotype correlation of hereditary enamel defect, and it is encouraged to screen *Sp6* gene in the of AI patients with unknown cause. Further studies on molecular basis of SP6 in amelogenesis could promote the development of the therapeutic application, such as gene therapy.

## **Conclusion**

We propose the addition of *Sp6* mutation in the AI classification as a new molecular criterion to properly diagnose and to apply it to development of therapeutic tools**.** Further analyses for the regulation of the temporospatial expression of SP6 and its function may clarify the mechanisms of AI disease and amelogenesis.

## **Abbreviations**

AI: Amelogenesis Imperfecta; Fst: Follistatin; SHR-SP: Stroke-prone Spontaneously Hypertensive Rat; Sp6: Specificity protein 6; TG: Transgenic.

## **Competing interests**

The authors declare no competing interests.

## **Authors’ contributions**

TM maintained rats, contributed to the experimental design, performed genotyping, western blot analysis, immunohistochemistry, histological analyses, macroscopic analysis, linkage analysis, X-ray analysis, and the manuscript preparation. KM maintained rats, contributed to the coordination of study, performed cDNA sequencing, western blot analysis, immunocytochemistry, and the manuscript preparation. TH maintained rats, and TH and HH performed cDNA sequencing. TN contributed to the project design and data interpretation, coordination of study, and the manuscript preparation. All authors read and approved the final manuscript.
